# Assessing the Distribution of Elderly Requiring Care: A Case Study on the Residents in Barcelona and the Impact of COVID-19

**DOI:** 10.3390/ijerph17207486

**Published:** 2020-10-15

**Authors:** Enrique Arvelo, Jesica de Armas, Monserrat Guillen

**Affiliations:** 1 Dept. of Economics and Business, Universitat Pompeu Fabra, 08005 Barcelona, Spain; enrique.arvelo@alum.upf.edu; 2 Dept. Econometrics, Universitat de Barcelona, 08034 Barcelona, Spain; mguillen@ub.edu

**Keywords:** elderly population, care system, COVID-19, data analytics

## Abstract

In this work, we establish a methodological framework to analyze the care demand for elderly citizens in any area with a large proportion of elderly population, and to find connections to the cumulative incidence of COVID-19. Thanks to this analysis, it is possible to detect deficiencies in the public elderly care system, identify the most disadvantaged areas in this sense, and reveal convenient information to improve the system. The methods used in each step of the framework belong to data analytics: choropleth maps, clustering analysis, principal component analysis, or linear regression. We applied this methodology to Barcelona to analyze the distribution of the demand for elderly care services. Thus, we obtained a deeper understanding of how the demand for elderly care is dispersed throughout the city. Considering the characteristics that were likely to impact the demand for homecare in the neighborhoods, we clearly identified five groups of neighborhoods with different profiles and needs. Additionally, we found that the number of cases in each neighborhood was more correlated to the number of elderly people in the neighborhood than it was to the number of beds in assisted living or day care facilities in the neighborhood, despite the negative impact of COVID-19 cases on the reputation of this kind of center.

## 1. Introduction

Caring for the elderly is a profoundly human experience. Throughout human history, every generation has been tasked with caring for their elderly when they reach their later years. Despite the universal nature of elderly care, it can be carried out in different ways. Apart from familial care, there are three main types of elderly care services: assisted living facilities (AL), day care facilities (DC), and homecare services (HC). All three services can be privately operated as a private for-profit business or publicly operated government programs. For example, in Barcelona, the local government is obliged to provide elder care services to all residents who cannot afford basic support. However, there are also higher-end alternatives for individuals who are willing and able to pay for them. From the point of view of companies or institutions offering any of these care services, it is important to balance the quality of service and the operating efficiency, although it is not easy to measure and quantify it [1]. All of these services are designed to fulfill the special needs of senior citizens, who can be defined as those above the age of 65. The eldercare market is an enormous global market, which is estimated to exceed 417 billion USD by the year 2026 [2].

AL, colloquially known as nursing homes, are a popular form of elderly care where the elderly live in dormitory-like facilities among some of their peers. In these institutions, the elderly citizens have around-the-clock immediate access to nurses who can assist them with their daily activities such as bathing, taking medicine, and cleaning. In these dwellings, the elderly are provided meals in common areas or rooms, and they have the opportunity to socialize with their co-residents, both informally and in organized activities. In some countries, other similar forms of congregant living arrangements have become popular, such as independent living and continuing care retirement communities [3]. In Spain, there are 5400 nursing homes, in which about 370,000 people live [4].

DC are similar to assisted living facilities where the elderly go to spend their days. Elderly clients are watched over and cared for. Then, every night, they each go back to their private homes, similar to how day care works for children. It is a popular option for elderly people who live with their family but they do not have the knowhow or time to take care of them during the day. The location of both AL and DC is relevant, and the benefits, opportunities, costs, and risks should be considered in this decision [5].

Homecare is an up-and-coming form of elderly care that has been gaining popularity in the last decade. Under a homecare system, individuals live at home and they are visited by skilled homecare workers who help them with health issues and with daily activities that they are no longer capable of doing themselves. The workers go from house to house, providing different services to different individuals throughout the day. As in AL and DC, we can differentiate between health and social care. Both are important, although it is difficult to measure the value of social care [6]. In HC, some of the many services provided by social homecare workers are bathing, cooking meals, buying groceries, cleaning the apartment, or taking them outside to get some fresh air or even visit doctors.

In the context of this study, we refer to care demanders as elderly living in a situation where they need help from a third person to develop their daily activities, and they cannot afford a private care service or family care at home. Thus, the aim of this study was to establish a methodological framework to analyze the care demand for elderly citizens in a certain area, and to find connections to the number of confirmed cases of COVID-19 before 20 July 2020. Thanks to this analysis, it is possible to detect deficiencies in the public elderly care system, identify the most disadvantaged areas in this sense, and reveal convenient information to improve the system. This is important, not only because the aging population is increasing in many regions of the world, but because today, it is expected that COVID-19-style pandemics will continue to occur, devastatingly affecting this population. Therefore, proper elderly care systems are key to avoiding bleak situations.

To the best of our knowledge, there is no other similar study on this topic. This is the reason why we established a methodological framework to apply this kind of study to any area with a large proportion of elderly population. As a case study, we focus on the city of Barcelona, where about 20% of residents are 65 years old or older (350,000 in 1.6 million residents) [7].

**Figure 1:**
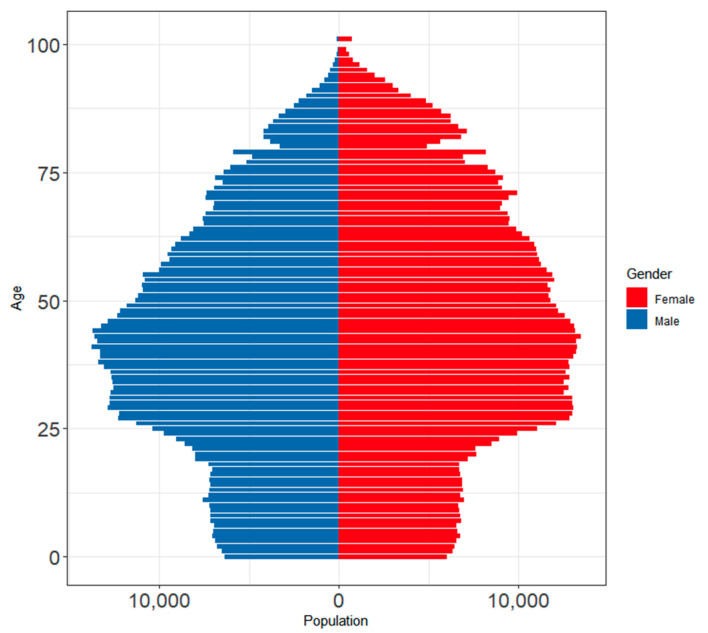
Barcelona’s population pyramid, 2020.

Along with some cultural changes in the last decades in Spain (decreasing percentages of multigenerational homes, increasing divorce rates among the elderly, etc.), it is predicted that a shift in demographics will lead to a significant increase in the demand for elder care services in the next couple of decades as the population of Barcelona ages. The population pyramid gives us a glimpse into what the future demographic of Barcelona may look like. Barcelona’s population pyramid (Figure 1) has a narrow base of younger people under 25, indicating a decline in the birthrate, which then dramatically widens out until the late 30s, which is where the bulk of the working population are, and then slowly narrows out every year. This type of tree indicates that Barcelona may face a demographic change in the near future and have a disproportionate elderly population as those in the middle age group and the bulge slowly work their way up the pyramid. Given this information, Barcelona must invest in the care system now, especially regarding services to the elderly and disabled, to meet the changing demand in the future. Taking it into account and considering how COVID-19 has impacted this city, Barcelona becomes a very good area to focus on as a case study.

Accordingly, this work is structured as follows. Section 2 is devoted to describing the data required and the methodology proposed to develop the analysis; in Section 3, we outline our tests and results; Section 4 provides a discussion on pros and cons of each kind of care service in Barcelona and demand trends in the future; and finally, conclusions and implications are described in Section 5.

## 2. Materials and Methods

To develop this study, we gathered some data about Barcelona from different sources, and we applied several analysis techniques to extract patterns and knowledge. In the following, we detail the data sources and variables and the methodology applied.

### 2.1. Data and Variables

A comprehensive database of Barcelona’s demographics was compiled primarily through combining several tables from Open Data BCN [8]. Open Data BCN is a public resource offered by the Barcelona government, where they host hundreds of high-quality open-source data tables. Barcelona’s AL and DC are also available in this data source. In addition to the data tables from Barcelona Open Data, there were some relevant data manually extracted from official government reports and papers published on Barcelona’s government website [9]. These data have been complemented with COVID-19 information. On July 16th 2020, the first wave of COVID-19 was virtually finished, with no cases in hospitals and almost no new cases per day. We compiled two indicators, the cumulated number of confirmed COVID-19 cases and the rate of confirmed cases per 100,000 residents by zone. The data were provided directly by the Municipal Data Office. Table 1 shows a summary of the data sources.

A few publicly available variables that would be interesting to analyze to gauge the demand for elderly care in Barcelona are the Total Population, RFID (Spanish acronym of disposable household income index), Elderly Population, Elderly Population Living Alone, and Disabled Elderly Population. The total population is a global variable for understanding the scale of the city. The RFID is a centered index that measures the disposable income per household, where neighborhoods with mean values are given an RFID index of 100. Given the current understanding, regions with higher disposable income will demand less public elderly care since they can afford more premium private services. The elderly population is a significant variable because they are the target audience, representing the total possible consumer population. Elderly Population Living Alone is a critical demographic for care services because this demographic does not have immediate familial support to help them complete daily activities. Finally, the disabled elderly population is another critical demographic. These individuals are elderly people suffering from some degree of disability that may impair them in completing everyday activities.

### 2.2. Methodology

We propose a methodological framework composed of a sequence of steps to analyze the demand for elderly care services in a predefined area and to find connections to the cumulative incidence of COVID-19. As mentioned before, we chose Barcelona due to the elderly population percentage and number of cases of COVID-19, although the same methodology can be applied to any other similar area. For the subsequent analysis, some analytic techniques were employed to reveal patterns and structure hidden within the data.

Choropleth Maps (CM) are used to understand the distribution of the elderly demanding care services across different neighborhoods by analyzing the distribution of variables linked to this demand.

Cluster Analysis (CA) serves to elucidate the elderly demographics across neighborhoods. These clusters then provide a framework for developing a plan for addressing the different demands of the neighborhood groups.

Principal Component Analysis (PCA) summarizes complex data with several variables into a lower dimension or a single figure, allowing one to understand the patterns in the data and how the variables relate to one another. In this study, PCA was used to understand where each neighborhood is positioned concerning select demographic variables.

Linear Regression (LR) is a classical statistical technique to model the expected outcome as a function of covariates. Here, we modelled the number of COVID-19 cases per zone and the number of confirmed cases per 100,000 residents as a function of some demographic characteristics. The aim of this analysis was to detect the most important factors in the scope of this work that impacted the number of COVID-19 cases.

## 3. Results

Our case study analyzed the area of Barcelona to understand the needs of care for Barcelona’s elderly population and to find connections between COVID-19 cases and some variables related to the elderly population. In the following sections, we detail the four framework steps applied to this case study.

### 3.1. Distribution of the Demand for Elderly Care Services in Barcelona Using CM

Through this analysis, we took a deeper dive into the distribution of the previously discussed demographic variables across Barcelona’s different neighborhoods. This allows us to begin to understand how the demand for elderly care is distributed across neighborhoods and how resources should be efficiently allocated. In the following, we analyze the distribution of the population in detail, considering the whole population, elderly population, RFID, elderly population living alone, and disabled elderly population.

- Population: The population of neighborhoods (see Figure S1a) has a positively skewed distribution, with a mean of 22,203 and a median of 20,487. The population has a wide range, ranging from 610 in *el Clot* (42) to 58,180 in *la Nova Esquerra de l’Eixample* (09). Looking at the map, it is clear that the distribution is not randomly distributed geographically. The bulk of the population is centered in the center of the city, in the *Eixample* neighborhoods and the neighborhoods immediately to the north of it. Apart from this central cluster, *Sant Andreu*, in the top right, has the second-highest population of any of the neighborhoods. The neighborhoods in the top of the map near the mountains and bottom of the map near the coast are less populated on average.
- Elderly Population: The total elderly population, intuitively, very closely resembles the distribution of the population (see Figure S1b,c). The mean elderly population is 4782.63 per neighborhood, ranging from 90 people in *el Clot* (42) to 13,413 in *la Nova Esquerra de l’Eixample* (09). To account for the difference in demand between the neighborhoods, the darker shaded neighborhoods should typically be allocated more resources than lighter shaded neighborhoods to allow them to service their larger elderly population. However, the number of resources must be adjusted to account for the RFID, elderly living alone, and disabled elderly population. The map of the relative percentage of elderly per neighborhood sheds some light on how the distribution of the elderly differs across different neighborhoods. The general geographic trend is that the neighborhoods tend to have a higher percentage of elderly as one moves up from the coast to the mountains.
- RFID: The RFID index (see Figure S1.d) has a wide range, where the most impoverished neighborhood is *la Trinitat Nova* (53), with an RFID index of 34.70, indicating that the average household in that neighborhood has 34.70% of the average disposable income. The wealthiest neighborhood is *Pedralbes* (21), with an RFID index of 251.7, indicating that the average household in that neighborhood has 2.5 times more disposable income than the mean. Like many income variables, it is very positively skewed, with the high-income area pulling the average up; 72.6% of the neighborhoods lie below the average, with only 27.3% lying above the average. This variable can be used as a scaling factor, where neighborhoods that have above the mean RFID receive fewer relative resources for their population. The map shows that the city’s income seems to be correlated with geographic location. The neighborhoods in the *Eixample*, *Sarrià*, *Gràcia*, and *Les Corts* tend to have RFID scores above 100. The five neighborhoods in the top of the map, *Trinitat Nova*, *Torre Baró*, *Ciutat Meridiana*, *Vallbona*, and *la Trinitat Vella*, and the neighborhood at the bottom, *la Marina del Prat Vermell*, have the lowest RFID scores, all below 50. Then, the majority of the neighborhoods on the right half of the map and the bottom left of the map have RFID indexes between 50 and 100.
- Elderly Population Living Alone: The elderly living alone map (see Figure S1e) correlates with the elderly population map; as the number of elderly increases, those that live alone will also increase. Therefore, to extract new information, it would be interesting to analyze the relative distribution of elderly people living alone (Figure S1f). The percentage of elderly living alone has a reasonably normal distribution with a mean of 25.07%. The variable ranges from 13.56% in *Vallbona* (56) to 34.34% in *Sant Pere*, *Santa Caterina i la Ribera* (04). There is not a clear geographic concentration or correlation like in the previous charts; however, we can still see some minor patterns. *Ciutadella* district neighborhoods have a large percentage of elderly people living alone. Like the RFID, this variable can be used to adjust the base demand. Neighborhoods with more significant percentages of elderly people living alone should receive a greater share of resources than a neighborhood of a similar population with a lower percentage of elderly people living alone, all other things held equal.
- Disabled Elderly Population: The base elderly disabled population stat is highly correlated with the elderly population (Figure S1g,h); therefore, we look at the percentage of elderly that are disabled in the neighborhood. The percentage of disabled, again, has a relatively normal distribution, with a mean of 20.41%. The variable ranges from 12.94% in *les Tres Torres* (24) to 33.80% in *la Marina del Prat Vermell* (12). The geographical pattern resembles the RFID pattern, but high income is negatively correlated with high disability rates. Once again, this variable can be used to scale the base demand for homecare, where neighborhoods with larger percentages of disabled elderly people should receive a greater share of resources than a neighborhood with a similar population and a lower percentage of disabled elderly people, all other things held equal.

### 3.2. Clustering of Barcelona’s Neighborhoods

This section builds on the analysis of Barcelona’s demographics, describes a brief market study, and segments Barcelona’s neighborhoods based on the characteristics that are likely to impact the demand for homecare in the neighborhoods. This analysis will provide us with a deeper understanding of how the demand for elderly care is dispersed throughout the city and how it can be used to create a generalized action plan to target each of the segments before specializing in the individual neighborhoods.

This segmentation was created using a non-hierarchal k-means Euclidean clustering method to group the neighborhoods into different clusters. The variables used in our clustering were the scaled values for the absolute values of the elderly population, RFID, percentage of the elderly with a disability, and percentage of elderly living alone. These variables were chosen because these variables directly impact the resources needed to serve the elderly of the community. The variables where then scaled (normalized) using a min–max scaling algorithm to account for the difference in the scale and variance of each variable. The scaling made it so that no variable dominated the other variables.

**Figure 2:**
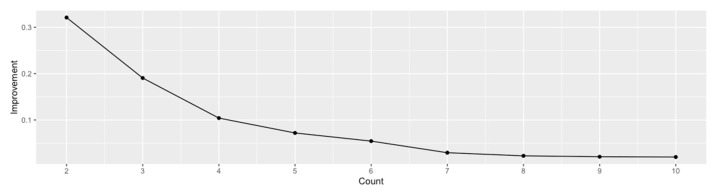
Increments in between-cluster variance.

The incremental improvement in the between-cluster variance for the Barcelona neighborhood data is shown in Figure 2. Given that we wanted to maximize the improvement while keeping the number of clusters relatively small, five clusters seemed to be the optimal number. Five clusters are a manageable number that allows for more straightforward interpretation and the development of unique strategies for each cluster of neighborhoods.

The between-cluster variance was equal to 0.687. This indicates that the clustering explained approximately 68.7% of the total variance in the data set. The neighborhoods were not evenly distributed among the clusters, with Cluster 1 with 13, Cluster 2 with 14, Cluster 3 with 7, Cluster 4 with 29, and Cluster 5 with 10 (see the outcome of CA in Table S1).

**Figure 3:**
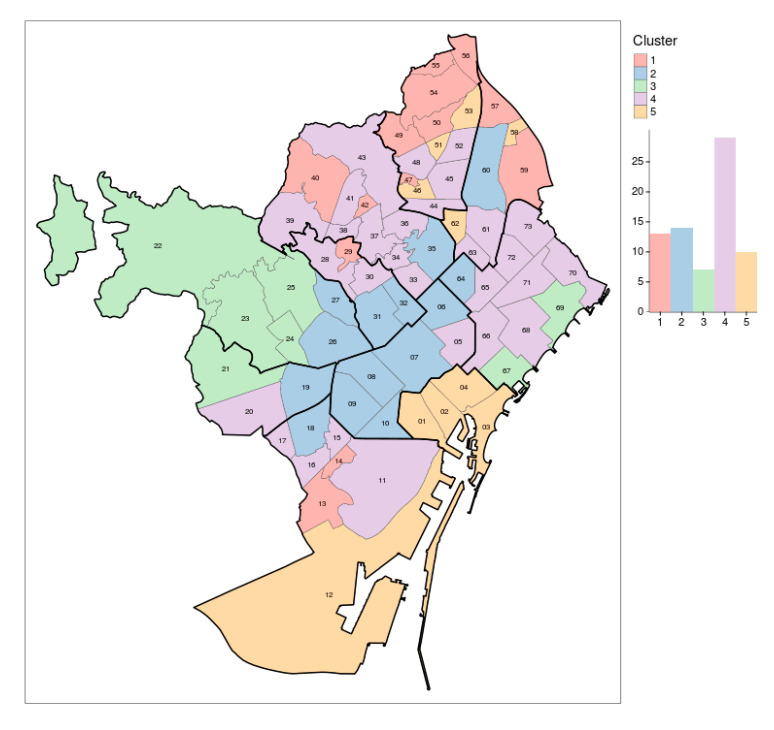
Maps of clusters.

These clusters were then mapped as shown in Figure 3, to better understand the make-up of the different neighborhood groups. Analyzing this figure, it is interesting to see how geographically similar the neighborhoods in each cluster were to one another. Cluster 1 is composed of outskirt neighborhoods on the borders of the city, primarily on the top right. Cluster 2 is composed of the neighborhoods in the center of the city. Cluster 3 is primarily composed of neighborhoods in the top left in the Sarria-Sant Gervasi district. Cluster 4 is the largest group with the largest spread, with the bulk of the neighborhoods spanning from the bottom right to the central top. Finally, Cluster 5 is composed of neighborhoods primarily in the Ciutat Vella district, with a few neighborhoods scattered in the top right. This map is useful for understanding the cluster’s general distribution and how the population in the city is distributed using one simple figure.

**Figure 4:**
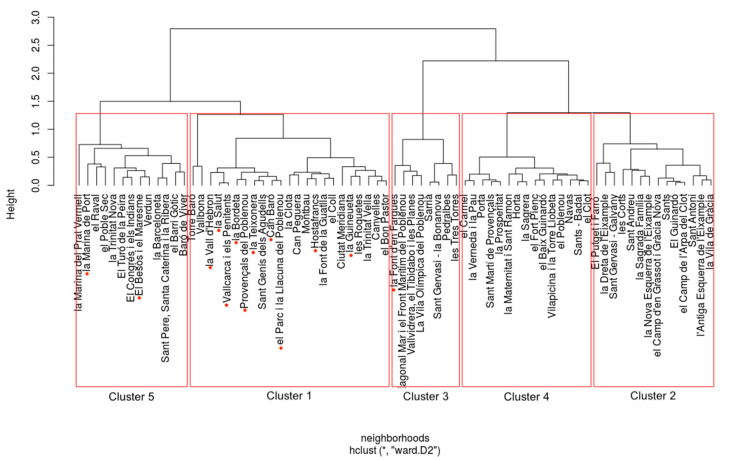
Robustness check dendrogram using Ward clustering.

The next step was to validate the robustness of the k-means clustering. The Ward clustering for the Barcelona data is presented in Figure 4.

As we can see from the dendrogram and Table 2andTable 3, the models were similar but not identical, with an overall hit rate of 82.3%. In other words, 82.3% of the observations from the initial k-means cluster were placed in the same cluster under the Ward clustering. Breaking it down by clusters, Cluster 1 had a hit rate of 92.3%, Cluster 2 had a hit rate of 100%, Cluster 3 had a hit rate of 100%, Cluster 4 had a hit rate 58.7%, and Cluster 5 had a hit rate of 100%. The majority of the misclassifications occurred between Clusters 1 and 4, where ten neighborhoods that were classified in Cluster 4 using k-means were classified in Cluster 1 when using the Ward distance.

These results were mixed. Clusters 2, 4, and 5 were fairly consistent across the two models, suggesting that these clusters are discrete structures in the data. However, the models had a difficult time distinguishing between Clusters 1 and 4. These two clusters had very similar distributions for the RFID and percentages of elderly people living alone, which caused some smaller neighborhoods in Cluster 4 to be classified as Cluster 1 using the Ward method. Looking forward, the PCA shows that although clusters were distinguishable, they bordered one another, with some neighborhoods in both clusters having similar characteristics. The largest distinguishable difference between these “borderline” neighborhoods is that those in Cluster 1 had a higher percentage of disabled elderly citizens.

The goal of the segmentation was to serve as an exploratory exercise to understand the clusters better and develop a general action plan for implementing care services in the different neighborhoods of Barcelona. This segmentation would need to be taken along with other analysis such as the maps or PCA. Therefore, although the segmentation was not entirely robust, its hit rate was tolerable for the scope of this work.

The distribution of the clusters for critical variables was analyzed (Figure S2 corresponds to four boxplots, which are the basis for the profiles of the different clusters).

- Cluster 1: Forgotten Neighborhoods. This cluster represents Barcelona “Forgotten” neighborhoods. Cluster 1, along with Cluster 4, is home to Barcelona’s most vulnerable elderly population. These small neighborhoods on the outskirts of the city have a low population, the lowest levels of disposable income, and high levels of disabled elderly. However, they, fortunately, have a lower percentage of elderly people living alone. It is imperative that despite the peripheral location and low population, these neighborhoods receive adequate resources. The low income and high disability indicate that neighborhoods in this cluster require a more substantial amount of resources per capita than Clusters 2, 3, and 4.
- Cluster 2: Densely Populated City Districts. This cluster represents the more stereotypical, highly populated neighborhoods. These neighborhoods have large populations with the overall largest number of elderly people. These districts have a high percentage of elderly living alone. However, this is offset by an above-average disposable income and low percentages of elderly people. These districts need a large number of resources to serve a large number of people in the neighborhoods. However, they likely need fewer resources per person due to their above-average wealth and low disability rate among the elderly.
- Cluster 3: Wealthy and Healthy. This cluster represents Barcelona’s most privileged communities. Cluster 3 has, by far, the highest disposable income, giving them much greater access to resources than others. This district has the lowest disability rate among the elderly and the lowest percentage of elderly living alone. This district would likely need fewer resources per elderly citizen than the rest of the districts. The high wealth will allow a possible user to afford more premium elder care services. The low percentage of elderly living alone indicates that they, on average, have support, which decreases the demand for homecare in minor cases. All this compounded with the fact that the district has the lowest elderly disability rate indicates that resources could be better used in other districts.
- Cluster 4: Middle Class. This cluster is the largest in terms of neighborhoods, and it represents Barcelona’s middle-income neighborhoods. This cluster of neighborhoods is the second most populous behind the cluster. They have a middle level of income, with an RFID around 76, which is around the midpoint when accounting for the right skew of the variable. When compared to the other neighborhoods, it has mid-level percentages of disabled, elderly, and disabled elderly. These neighborhoods act as the baseline for Barcelona in terms of demand for care services. They should receive more resources per capita than Clusters 2 and 3 but less than Clusters 1 and 5.
- Cluster 5: Vulnerable. This cluster represents Barcelona’s most vulnerable neighborhoods. These neighborhoods, along with those in Cluster 1, have the lowest disposable income in Barcelona. In addition, these neighborhoods have, by far, the highest percentages of elderly living alone and of elderly with disabilities. Putting it all together, it paints a grim picture for the quality of life for the elderly citizens in this cluster. These neighborhoods should be a priority when implementing an elderly care system in Barcelona and be allocated the highest amount of resources per capita compared to the rest of the clusters.

### 3.3. PCA of Barcelona’s Neighborhoods

PCA is an additional tool that, along with the segmentation and CM, can help to characterize the different neighborhoods in terms of some key variables. The directions of the arrows represent the directions in the plot that the particular variables increase, and the color of the text represents the cluster of the neighborhood. Overall, the PCA was able to capture 78% of the data’s variation, indicating that the chart is useful but should not be taken as an exact representation.

In a PCA analysis, it is possible to form a characterization of a neighborhood or group of neighborhoods by looking at where they are positioned in the plane and the direction of the arrows (see Figure S3).

At an initial glance, the chart seems to support the previous CA and their characterizations. Except for a few outliers, the clusters lie in clear groups. These characterizations created with the PCA corroborate those developed with the initial CA.

- Cluster 1 is spread out into the top right region of the map; we can see that neighborhoods in this cluster tend to have a smaller cumulative population of elderly citizens, a lower percentage of people living alone, a higher percentage of disabled, and a lower RFID index.
- Cluster 2 is located at the bottom left of the chart; this positioning indicates that these neighborhoods have a higher cumulative number of elderly citizens, a higher than average RFID, a large percentage of elderly citizens living alone, and a low percentage of disabled elderly people.
- Cluster 3 is located on the top left of the map, indicating that it has the highest RFID, a somewhat higher number of elderly than average, low percentages of elderly people living alone, and the lowest percentage of disabled elderly.
- Cluster 4, as mentioned in the previous section, represents the median neighborhoods, as it lies in the midpoint of the chart, indicating it represents the mean value for the four variables.
- Cluster 5 is located on the bottom right of the map. Like Cluster 1, it has lower values for the RFID and a high percentage of disabled elderly. However, unlike Cluster 1, it is located towards the bottom of the map, indicating a large elderly population and a large percentage of elderly people living alone.

### 3.4. Analysis of Barcelona’s COVID-19 Cases

Table 4 presents the results of regression models for the number of COVID-19 cases in each neighborhood as a function of some characteristics. The results show that the most important factor is the number of elderly residents in the neighborhood. What we can obtain from such a model is an association; we cannot really prove causality, but we can conclude that there is a positive relationship between those zones with a higher number of elderly people and those zones with a higher number of COVID-19 cases. Once this demographic variable is in the model, the number of AL or DC does not seem to be related to the outbreak. We may also add that younger people have had a tendency to be carrying the virus while experiencing no symptoms themselves, whereas elderly people showed more severe signs and so they were tested and confirmed. At the current stage, we cannot see a causal relationship, but we certainly see that the COVID-19 outbreak was not exclusive to community services where elderly people live together, even if contagion between elderly patients was easier in those institutions than at home.

In order to see the influence of service capacity and the percentage of disabled elderly citizens on the response variable, which is the number of cases in the neighborhood after the first wave, we created eight different models. Table 4 displays, in the first four columns, the models that do not include the percentage of disabled elderly residents and, in the last four columns, the models that contain this covariate. In models (1) to (4) and (5) to (8), we introduce the DC capacity, AL capacity, total elderly service capacity, and former two. The model results show adjusted R^2^ above 75%, and all the models indicate that the larger the number of elderly residents, the higher the expected number of total cases in the neighborhood, while the higher the income index (RFID), the lower the expected number of cases in the neighborhood; i.e., wealthier areas are expected to have fewer cases.

In order to provide a comparison that is not influenced by the population size in each neighborhood, we analyzed COVID-19 cases per 100,000 residents. In this new set of models, we included the percentage of elderly population instead of the total elderly population. Table 5 presents the model results. The results in Table 5 lead to the same conclusions as those in Table 4; the expected number of COVID-19 cases per 100,000 residents increases in neighborhoods with a larger percentage of elderly population and lower income index, while there is no evidence of an association with the number of elderly services (AL, DC, or both) in the neighborhood.

**Figure 5:**
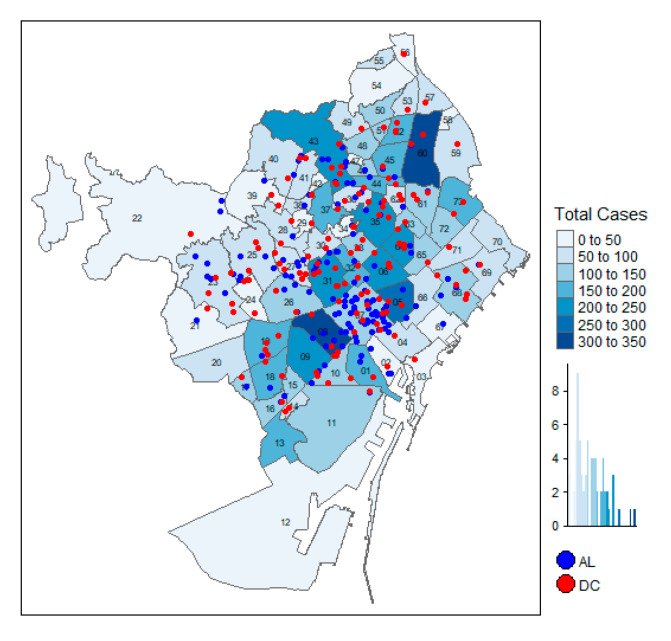
Location of AL/DC and total cases.

**Figure 6:**
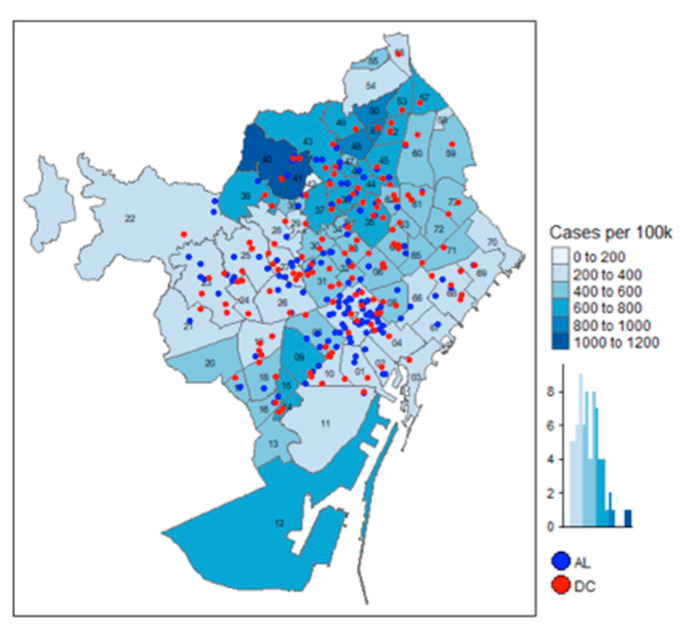
Location of AL/DC and cases per 100,000.

Figure 5 and Figure 6 show that the density of COVID-19 cases is not concentrated in the areas with more facility services, but it could be argued that the centers in the central areas are smaller than those located in the outskirts. The regression models control for the size of the AL and DC services, and they show that, when controlling for the influence of the elderly residents, AL and DC size are not associated with a larger number of expected positive COVID-19 cases.

## 4. Discussion

As mentioned before, there are three main kinds of care services for elderly people, AL, DC, and HC. Additionally, teleassistance and similar options seem to have gained popularity during the first coronavirus wave [11]. These are offered to fragile citizens who live alone or are alone most of the day. Citizens can obtain urgent assistance by simply pushing a button in a necklace, or they can be contacted by phone.

Within this global vision of elderly care services, the advantages of HC can be summarized in the following main ideas: lower cost and more scalable than other options without decreasing care quality, increased independence of the elderly person, better quality of life, and limited contact between elderly individuals, which implies limited spread of viral diseases in the case of an outbreak. A study by Nordic researchers stated that “On average, a place in a retirement home costs 49,500 euros annually, whereas homecare costs 20,300 euros” [12]. These savings show that expanding the homecare system is generally more cost-effective than systematically moving individuals into assisted living facilities.

Breaking it down, to implement an assisted living center or a day center, it is necessary to build a physical facility with room to house patients, common areas, dining areas, bathrooms, and kitchens. These facilities would cost a significant amount of money to set up and have significant maintenance costs associated with cleaning and rent. Additionally, as the number of patients admitted to the facilities increases, the quality of service each client receives decreases as the relative space per user drops [13,14]. Each facility has a maximum capacity, and once that capacity is met, they cannot accept any more people. This leads to scenarios of underserved communities, where people needing care are denied state-run AL and put on long waitlists with no guarantee of ever getting off [15].

By nature of their design, HC avoid many of these problems, because elderly people stay in their own homes and if demand increases, providers may recruit additional specialized carers [16]. Indirect savings from homecare come from a decreased number of doctor visits and hospital admissions for people enrolled in homecare systems. A 2014 study found that elderly residents enrolled in HC who had experienced some mental decline had a significantly lower inpatient hospital admission rate than those without HC [17]. They also went to the doctor 25% less than those who were not enrolled [18]. These studies show that adopting HC can lead to significant savings later on.

Another critical reason to adopt HC is that, in general, elderly people prefer them to the other options. HC provide the user with a level of autonomy, comfort, and privacy that is not available from the other options. A 2016 survey conducted by the American Association of Retired Persons (AARP) found that 90% of elderly citizens in the United States want to remain living in their homes for as long as possible [19].

The most topical reason for the adoption of HC is the reduction of disease spread. The layouts of AL and DC are conducive to the spread of viruses. In these facilities, the residents spend the vast majority of time under the same roof as dozens of other residents. They all share bathrooms, eat in dining halls, and occupy the same common spaces. If a visitor, worker, or doctor gets sick and passes the infection on to a resident, everyone else in the facility is at risk, and due to the design plan, it is difficult to maintain social distance and avoid contaminated surfaces when many people are living under the same roof [20]. This is increasingly worrisome when considering that elderly citizens who need assistance are the population group most vulnerable to some diseases such as the flu or viruses.

This problem was highlighted during the coronavirus crisis. The World Health Organization stated in mid-April 2020 that up to half of the COVID-19 deaths in Europe occurred in long-term-care facilities [21]. These centers facilitate the spread of disease and are not equipped to handle an outbreak once it occurs, although some policies have been considered after this first coronavirus wave [22]. The workers are often not medical professionals and do not feel the same obligation to treat their residents as a nurse or doctor would. Around the globe, family visits were banned to attempt to mitigate the spread of disease into nursing homes; however, this decision may have come with the unintended effect of many residents being forcefully detached from any support system and abandoned. A particularly gruesome example of this happened when the Spanish military was sent to disinfect AL around Madrid; they found dozens of residents either dead in their beds or left completely abandoned. This is not an isolated incident, as a large number of AL in Italy, Spain, and the United States are under investigation for malpractice. Unfortunately, this was a problem long before COVID-19, since studies have shown that AL are hotbeds for respiratory and gastrointestinal infections, although these devastating consequences often go unrecognized and unappreciated [23]. 

This grave problem could be mitigated by increasing the use of HC as the preferred method of elderly care. In a homecare environment, there are substantially fewer opportunities for contagion from co-residents. Users live in their own private homes and only come in contact with their homecare worker. For a disease to spread from one user to another, it would need to go from the infected person to the homecare worker and to the other, which is unlikely if proper safety precautions are taken such as washing hands, covering coughs, and using appropriate PPE when working with a sick patient. Additionally, homecare users will not lose access to their support systems during times of crises, as they have the autonomy to reach out for help from family, friends, or neighbors.

We anticipate that citizens would possibly prefer HC in the future due to the large number of COVID-19 cases observed during the first wave in AL. This has had an enormous negative impact on the reputation of AL. In this regard, we show that the number of cases in each neighborhood is more correlated to the number of elderly people in the neighborhood than it is to the number of beds in AL or to the number of DC in the area we have used as a case study.

## 5. Conclusions

Considering that the elderly population is on the rise in many countries of the world, there is a clear need for an adequate system to deal with the care of the elderly in the near future, especially in the face of pandemics such as COVID-19. Thanks to the methodological framework proposed in this work, it is possible to analyze the care demand for elderly citizens and to find connections to the number of cases of COVID-19 in any area with a large proportion of elderly population. As a case study, we applied it to Barcelona, a city with a large elderly population and a high incidence of COVID-19.

Thus, we have evidenced that data on elderly care services and demographic information help to understand the geographical dispersion and singularities of a large metropolitan area, showing that there may be zones more deprived than others and areas that need to improve the number of care services in the next decades. It is possible to detect deficiencies in the elderly care system and provide interesting insights to improve it. This analysis can be considered as a system to alert one to the need for better resources.

Additionally, our case study for the city of Barcelona, at the end of the first coronavirus wave of the 2020 outbreak, shows that the cumulative incidence of COVID-19 was higher in those geographical zones that had a large number of elderly citizens. This leads us to conclude that not only should AL and DC be under lockdown primarily in the early stages of an outbreak, but also that extended HC should be specially designed to isolate the fragile population at home, thus preventing contagion. Improving HC is especially important for active residents who do not need round-the-clock surveillance. 

We propose that teleassistance, which has a virtually 100% coverage of the target population at risk, elderly and fragile individuals living alone, can be used to provide these citizens with basic needs and support to avoid their contact with the community outside their dwellings.

As future work, we plan to monitor new waves of the coronavirus crises and their impact on the elderly population living in metropolitan areas. Since HC seem to be the preferred form of long-term care for the majority of citizens and they could be more demanded after this pandemic, we will work to propose a framework to assess the state of this kind of care system in a certain area. This is a challenge, since we will need data on elderly under this kind of care, their satisfaction, and the resources and cost of HC in that area. Depending on the particular area or country, HC can be provided by different institutions (health or social), increasing the difficulties in accessing and evaluating data about these services.

## Figures and Tables

**Table 1:** Data sources.

Index	Data Table	Source
1	Neighborhood population by gender	Open Data BCN
2	Neighborhood population by age	Open Data BCN
3	Neighborhood population by age quantiles	Open Data BCN
4	Neighborhood population by gender and age	Open Data BCN
5	Neighborhood disabled population by age quantiles	Open Data BCN
6	Neighborhood population living alone by age	Open Data BCN
7	Censual Districts population demographics	Open Data BCN
8	Neighborhood RFID index	Government Report
9	Geographic outlines	GitHub [10]
10	Assisted living facilities	Open Data BCN
11	Day care centers	Open Data BCN
12	COVID-19	Barcelona Municipal Data Office

**Table 2:** Misclassifications.

K-Means	Ward	n
One	Five	1
Four	One	10
Four	Three	1
Four	Five	1

**Table 3:** Hit rate.

Cluster	Hit Rate	
Overall	60/73	82.2%
One	12/13	92.3%
Two	14/14	100%
Three	7/7	100%
Four	17/29	58.7%
Five	10/10	100%

**Table 4:** Regression analysis for the number of confirmed cases of COVID-19 in Barcelona by neighborhood until 20 July 2020 (standard errors are displayed in parenthesis under the coefficient estimates, n = 73 neighborhoods).

Variable	(1)	(2)	(3)	(4)	(5)	(6)	(7)	(8)
Elderly Population	0.021 ^1^	0.021 ^1^	0.021 ^1^	0.021 ^1^	0.021 ^1^	0.021 ^1^	0.021 ^1^	0.021 ^1^
	(0.002)	(0.001)	(0.001)	(0.002)	(0.002)	(0.001)	(0.002)	(0.002)
RFID	−0.401 ^1^	−0.415 ^1^	−0.414 ^1^	−0.413 ^1^	−0.508 ^1^	−0.519 ^1^	−0.516 ^1^	−0.520 ^1^
	(0.101)	(0.105)	(0.105)	(0.106)	(0.13)	(0.133)	(0.133)	(0.134)
% of Disabled Elderly					−1.85	−1.765	−1.763	−1.856
					(1.427)	(1.416)	(1.417)	(1.435)
DC Capacity	−0.026			−0.044	−0.047			−0.066
	(0.125)			(0.133)	(0.125)			(0.134)
AL Capacity		0.006		0.008		0.005		0.008
		(0.019)		(0.02)		(0.019)		(0.02)
Total Facility Capacity			0.005				0.003	
			(0.018)				(0.018)	
Constant	45.544 ^1^	46.367 ^1^	46.273 ^1^	46.282 ^1^	96.024 ^2^	94.584 ^2^	94.417 ^2^	96.947 ^2^
	(10.864)	(11.013)	(11.018)	(11.088)	(40.404)	(40.207)	(40.211)	(40.715)
Observations	73	73	73	73	73	73	73	73
R^2^	0.785	0.785	0.785	0.785	0.79	0.79	0.79	0.791
Adjusted R^2^	0.775	0.776	0.776	0.773	0.778	0.777	0.777	0.775
Residual Std. Error	36.157 (df = 69)	36.144 (df = 69)	36.151 (df = 69)	36.380 (df = 68)	35.980 (df = 68)	36.000 (df = 68)	36.008 (df = 68)	36.201 (df = 67)
F Statistic	83.882 ^1^ (df = 3; 69)	83.960 ^1^ (df = 3; 69)	83.919 ^1^ (df = 3; 69)	62.183 ^1^ (df = 4; 68)	63.953 ^1^ (df = 4; 68)	63.864 ^1^ (df = 4; 68)	63.827 ^1^ (df = 4; 68)	50.572 ^1^ (df = 5; 67)

^1^*p* < 0.01; ^2^ *p* < 0.05.

**Table 5:** Regression analysis for the cases per 100,000 residents in Barcelona by neighborhood until 20 July 2020 (standard errors are displayed in parenthesis under the coefficient estimates, n = 73 neighborhoods).

Variable	(1)	(2)	(3)	(4)	(5)	(6)	(7)	(8)
% of Elderly	27.360 ^1^	25.544 ^1^	25.679 ^1^	26.504 ^1^	26.491 ^1^	25.028 ^1^	25.202 ^1^	25.428 ^1^
	(4.194)	(4.168)	(4.202)	(4.211)	(4.821)	(4.868)	(4.885)	(4.846)
RFID	−2.065 ^1^	−2.286 ^1^	−2.260 ^1^	−2.223 ^1^	−2.205 ^1^	−2.366 ^1^	−2.335 ^1^	−2.397 ^1^
	(0.383)	(0.397)	(0.4)	(0.397)	(0.538)	(0.555)	(0.555)	(0.552)
% of Disabled Elderly					−2.27	−1.252	−1.174	−2.768
					(6.084)	(5.986)	(6.006)	(6.052)
DC Capacity	−0.316			−0.585	−0.343			−0.623
	(0.405)			(0.446)	(0.414)			(0.457)
AL Capacity		0.062		0.106		0.062		0.108
		(0.069)		(0.076)		(0.069)		(0.077)
Total Facility Capacity			0.045				0.045	
			(0.064)				(0.064)	
Constant	120.975	155.419	151.546	145.196	199.628	199.332	192.58	241.573
	(95.199)	(96.341)	(96.774)	(96.16)	(231.587)	(231.256)	(231.475)	(231.876)
Observations	73	73	73	73	73	73	73	73
R^2^	0.532	0.534	0.532	0.545	0.533	0.534	0.532	0.547
Adjusted R^2^	0.512	0.514	0.511	0.519	0.506	0.507	0.504	0.513
Residual Std. Error	136.391 (df = 69)	136.186 (df = 69)	136.494 (df = 69)	135.482 (df = 68)	137.250 (df = 68)	137.140 (df = 68)	137.456 (df = 68)	136.277 (df = 67)
F Statistic	26.190 ^1^ (df = 3; 69)	26.338 ^1^ (df = 3; 69)	26.116 ^1^ (df = 3; 69)	20.389 ^1^ (df = 4; 68)	19.432 ^1^ (df = 4; 68)	19.491 ^1^ (df = 4; 68)	19.323 ^1^ (df = 4; 68)	16.163 ^1^ (df = 5; 67)

^1^*p* < 0.01.

## Supplementary Materials

The following are available online at https://www.mdpi.com/1660-4601/17/20/7486/s1. Figure S1: Choropleth maps, Figure S2: Boxplots of critical variables by clusters, Figure S3: PCA of the neighborhoods, Figure S4: Correlation matrix for data on 73 neighborhoods in Barcelona, Table S1: Cluster with k-means (k = 5). The database created and used in this work is available online at www.upf.edu/web/ephocas/repository.

## References

[1] Lin J.-R., Chen C.-Y., Peng T.-K. (2017). Study of the relevance of the quality of care, operating efficiency and inefficient quality competition of senior care facilities. J. Environ. Res. Public Health.

[2] Market Watch Elder Care Services Market Size 2020 Industry Share, Trends Evaluation, Global Growth, Recent Developments, Latest Technology, CAGR of 2.4%, and 2026 Future Forecast Research Report. https://www.marketwatch.com/press-release/elder-care-services-market-size-2020-industry-share-trends-evaluation-global-growth-recent-developments-latest-technology-cagr-of-24-and-2026-future-forecast-research-report-2020-07-14.

[3] Coe N.B., Van Houtven C.H. (2020). Living Arrangements of Older Adults and COVID-19 Risk: It Is Not Just Nursing Homes. J. Am. Geriatr. Soc..

[4] The New York Times A Deluged System Leaves Some Elderly to Die, Rocking Spain’s Self-Image. www.nytimes.com/2020/03/25/world/europe/Spain-coronavirus-nursing-homes.html.

[5] Lee A.H.I., Kang H.-Y. (2019). A multi-criteria decision-making model for evaluating senior daycare center locations. J. Environ. Res. Public Health.

[6] Burge P., Netten A., Gallo F. (2010). Estimating the value of social care. J. Health. Econ..

[7] Ajuntament de Barcelona Population Register 2019. https://www.bcn.cat/estadistica/castella/dades/tpob/pad/padro/evo/ev02.htm.

[8] Open Data BCN Ajuntament de Barcelona’s Open Data Service. https://opendata-ajuntament.barcelona.cat/en.

[9] Ajuntament de Barcelona Territorial Distribution of Family Income per Capita in Barcelona. https://ajuntament.barcelona.cat/barcelonaeconomia/ca/renda-familiar/renda-familiar/distribucio-territorial-de-la-renda-familiar-disponible-capita.

[10] Github Barcelona Geodata. https://github.com/martgnz/bcn-geodata.

[11] Portnoy J., Waller M., Elliott T. (2020). Telemedicine in the Era of COVID-19. J. Allergy. Clin. Immuno..

[12] Eveborn P., Rönnqvist M., Einarsdóttir H., Eklund M., Lidén K., Almroth M. (2009). Operations Research Improves Quality and Efficiency in Home Care. Interfaces.

[13] Tran A., Nguyen K.-H., Gray L., Comans T. (2019). A systematic literature review of efficiency measurement in nursing homes. Int. J. Environ. Res. Public Health.

[14] Garavaglia G., Lettieri E., Agasisti T., Lopez S. (2011). Efficiency and quality of care in nursing homes: An Italian case study. Health Care Manag. Sci..

[15] Foston Europe Senior Care in Spain. https://www.foston.eu/senior-care-in-spain/.

[16] Mills W.R., Buccola J.M., Sender S., Lichtefeld J., Romano N., Reynolds K., Price M., Phipps J., White L., Howard S. (2020). Home-Based Primary Care Led-Outbreak Mitigation in Assisted Living Facilities in the First 100 Days of Coronavirus Disease 2019. J. Am. Med. Dir. Assoc..

[17] Home Instead Senior Care and Global Coalition on Aging Relationship-Based Care and Positive Outcomes for People with Alzheimer’s and their Families. https://www.ncgg.go.jp/topics/dementia/documents/Topic1-7JeffHuber.pdf.

[18] Home Instead Senior Care Paid In-Home Care: More Care & Better Care for Seniors. https://www.homeinstead.com/Documents/BETTER%20CARE%20FOR%20SENIORS.pdf.

[19] National Conference of State Legislatures and AARP Public Policy Institute Aging in Place: A State Survey of Livability Policies and Practices. https://assets.aarp.org/rgcenter/ppi/liv-com/aging-in-place-2011-full.pdf.

[20] Dobbs D., Peterson L., Hyer K. (2020). The Unique Challenges Faced by Assisted Living Communities to Meet Federal Guidelines for COVID-19. J. Aging Soc. Policy.

[21] The Washington Post Nursing Homes Linked to up to Half of Coronavirus Deaths in Europe, WHO Says. https://www.washingtonpost.com/world/europe/nursing-homes-coronavirus-deathseurope/2020/04/23/d635619c-8561-11ea-81a3-9690c9881111_story.html.

[22] American Geriatrics Society (AGS) (2020). Policy Brief: COVID-19 and Assisted Living Facilities. J. Am. Geriatr. Soc..

[23] Strausbaugh L.J., Sukumar S.R., Joseph C.L., High K.P. (2003). Infectious Disease Outbreaks in Nursing Homes: An Unappreciated Hazard for Frail Elderly Persons. Clin. Infect. Dis..

