# Supplementary material for: Assessing the Distribution of Elderly Requiring Care: A Case Study on the Residents in Barcelona and the Impact of COVID-19
Source: Int J Environ Res Public Health. 2020 Oct 15;17(20):7486. doi: 10.3390/ijerph17207486 (PMC7602505; doi:10.3390/ijerph17207486)
Supplement: Supplementary file 1 [file ijerph-17-07486-s001.zip › Figure S1.docx]

**Figure S1: Choropleth Maps**

| 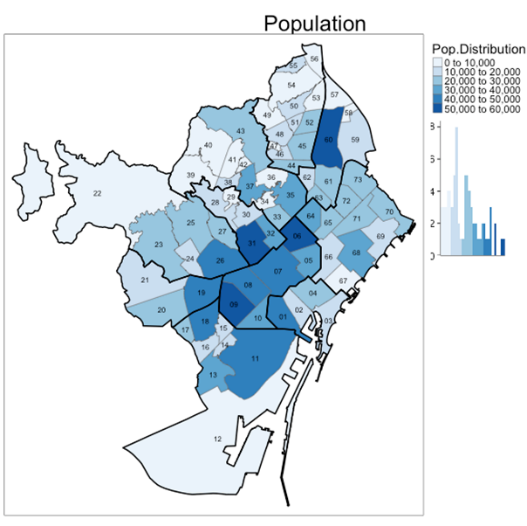  **Figure S1.a.** Population | 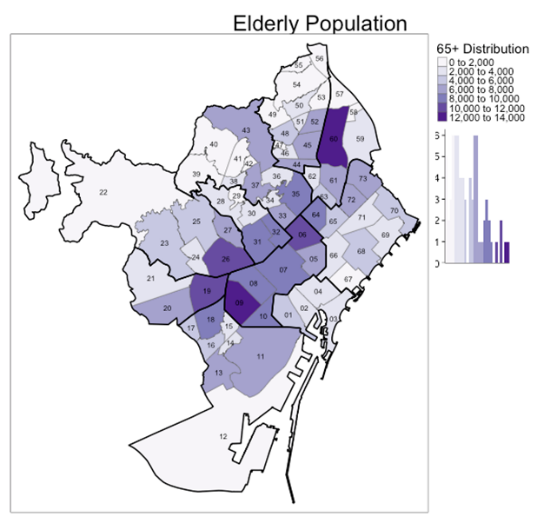  **Figure S1.b.** Elderly population |
| --- | --- |
| 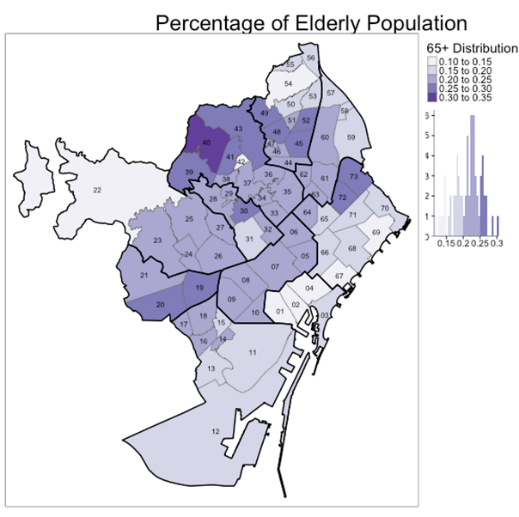  **Figure S1.c.** Percentage of elderly population | 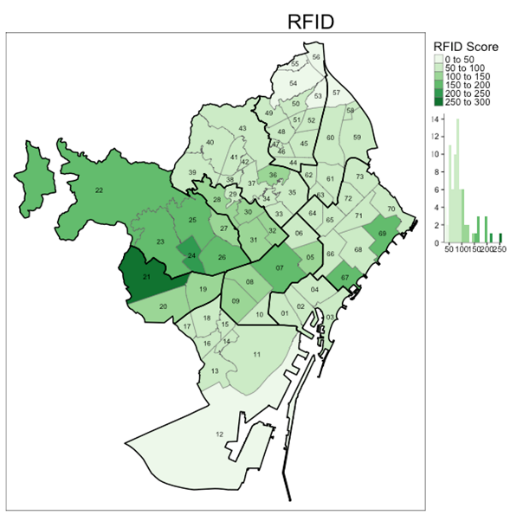  **Figure S1.d.** RFID |
| 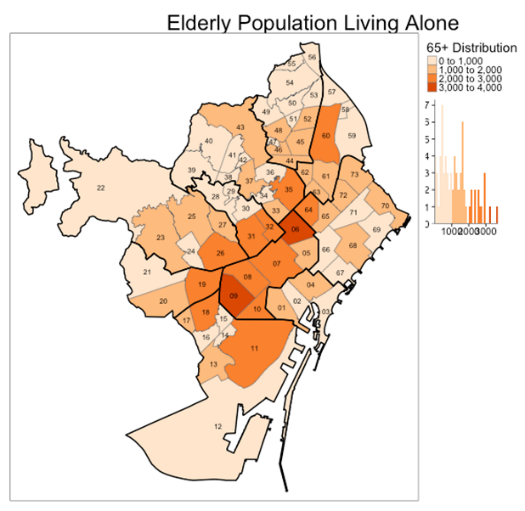  **Figure S1.e.** Elderly population living alone | 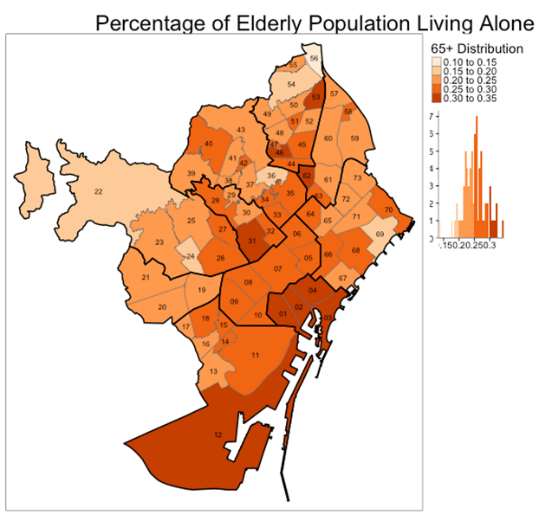  **Figure S1.f.** Percentage of elderly population living alone |
| 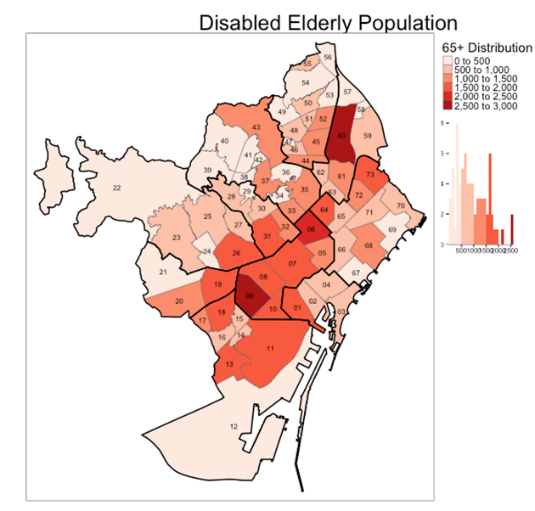  **Figure S1.g.** Disabled elderly population | 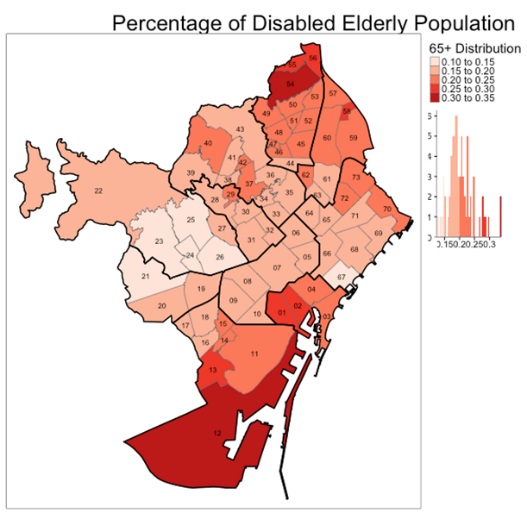  **Figure S1.h.** Percentage of disable elderly population |
