# Supplementary material for: Assessing the Distribution of Elderly Requiring Care: A Case Study on the Residents in Barcelona and the Impact of COVID-19
Source: Int J Environ Res Public Health. 2020 Oct 15;17(20):7486. doi: 10.3390/ijerph17207486 (PMC7602505; doi:10.3390/ijerph17207486)
Supplement: Supplementary file 1 [file ijerph-17-07486-s001.zip › Figure S2.docx]

**Figure S2. Boxplots of critical variables by clusters**


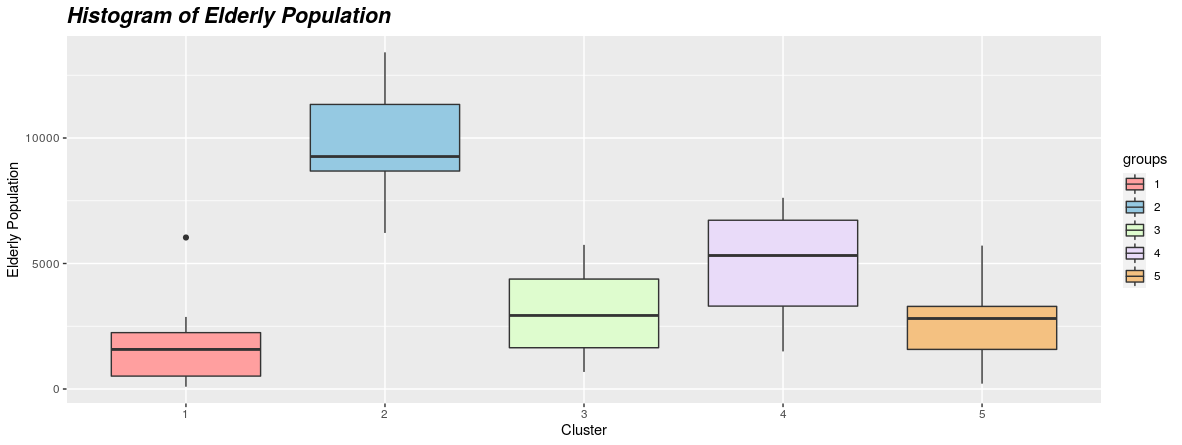


**Figure S2.a.** Boxplot of variable Elderly Population by clusters


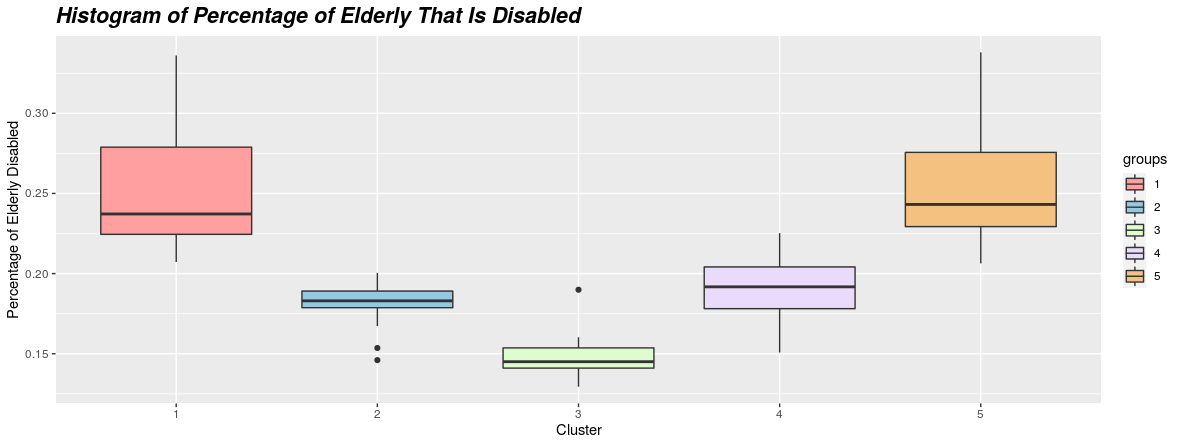


**Figure S2.b.** Boxplot of variable Elderly Disabled by clusters


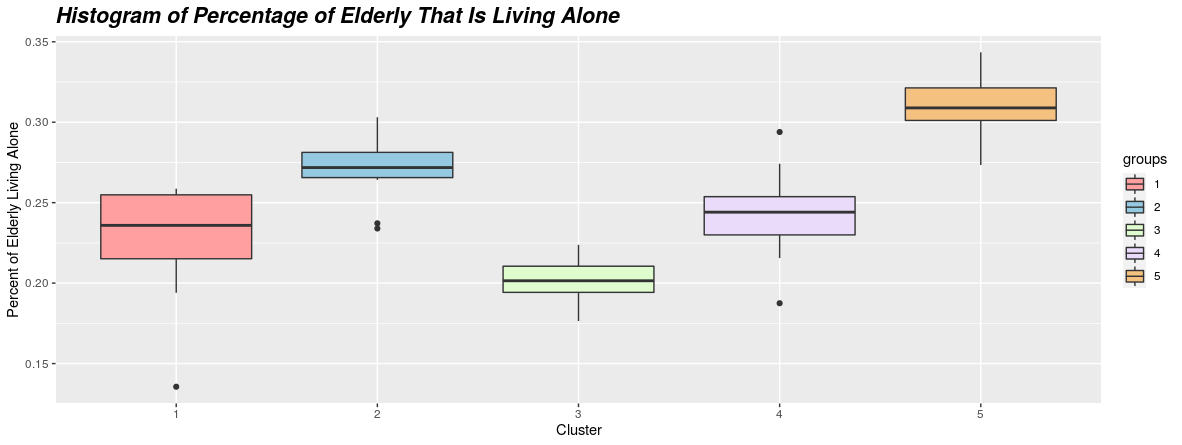


**Figure S2.c.** Boxplot of variable Elderly Living Alone by clusters


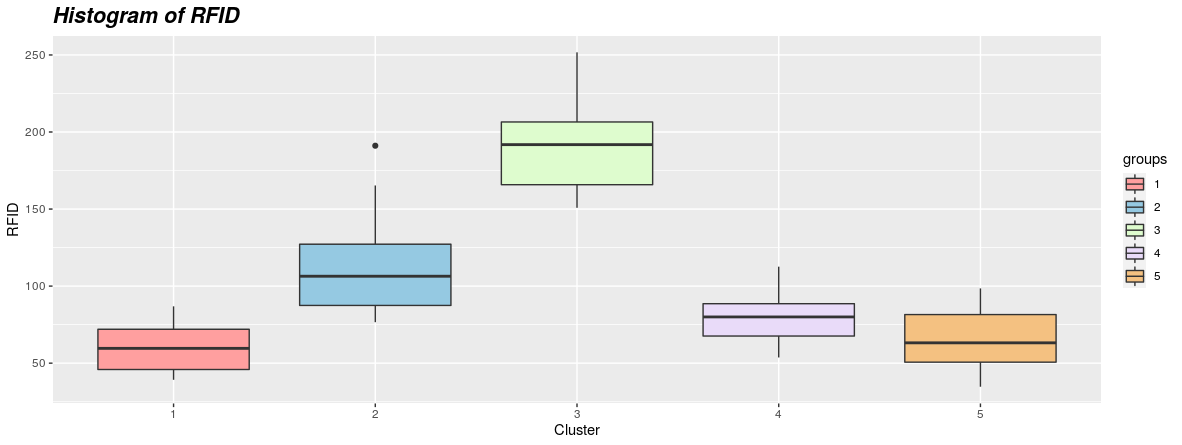


**Figure S2.d.** Boxplot of variable RFID by clusters
