# Supplementary material for: Assessing the Distribution of Elderly Requiring Care: A Case Study on the Residents in Barcelona and the Impact of COVID-19
Source: Int J Environ Res Public Health. 2020 Oct 15;17(20):7486. doi: 10.3390/ijerph17207486 (PMC7602505; doi:10.3390/ijerph17207486)
Supplement: Supplementary file 1 [file ijerph-17-07486-s001.zip › Table S1 .docx]

**Table S1. Cluster with k-means (k=5)**

| # | Cluster 1 | Cluster 2 | Cluster 3 | Cluster 4 | Cluster 5 |
| --- | --- | --- | --- | --- | --- |
| 1 | la Trinitat Vella | la Sagrada Família | Pedralbes | el Fort Pienc | el Raval |
| 2 | la Font de la Guatlla | la Dreta de l'Eixample | Vallvidrera, el Tibidabo i les Planes | el Poble Sec | el Barri Gòtic |
| 3 | la Marina de Port | l'Antiga Esquerra de l'Eixample | Sant Gervasi - la Bonanova | la Salut | la Barceloneta |
| 4 | Ciutat Meridiana | la Nova Esquerra de l'Eixample | La Vila Olímpica del Poblenou | el Baix Guinardó | Sant Pere, Santa Caterina i la Ribera |
| 5 | la Clota | el Camp de l'Arpa del Clot | Diagonal Mar i el Front Marítim del Poblenou | el Parc i la Llacuna del Poblenou | la Marina del Prat Vermell |
| 6 | Can Peguera | el Camp d'en Grassot i Gràcia Nova | Sarrià | Vilapicina i la Torre Llobeta | El Turó de la Peira |
| 7 | Canyelles | Sant Gervasi - Galvany | les Tres Torres | Sant Genís dels Agudells | El Congrés i els Indians |
| 8 | les Roquetes | El Guinardó |  | la Teixonera | Verdun |
| 9 | Torre Baró | Sants |  | la Vall d'Hebron | la Trinitat Nova |
| 10 | Montbau | les Corts |  | la Font d'en Fargues | Baró de Viver |
| 11 | Vallbona | El Putget i Farró |  | Horta |  |
| 12 | el Coll | Sant Andreu |  | el Carmel |  |
| 13 | el Bon Pastor | la Vila de Gràcia |  | Porta |  |
| 14 |  | Sant Antoni |  | Hostafrancs |  |
| 15 |  |  |  | la Bordeta |  |
| 16 |  |  |  | Sants - Badal |  |
| 17 |  |  |  | la Maternitat i Sant Ramon |  |
| 18 |  |  |  | Vallcarca i els Penitents |  |
| 19 |  |  |  | la Sagrera |  |
| 20 |  |  |  | Navas |  |
| 21 |  |  |  | el Clot |  |
| 22 |  |  |  | Can Baró |  |
| 23 |  |  |  | el Poblenou |  |
| 24 |  |  |  | El Besòs i el Maresme |  |
| 25 |  |  |  | Provençals del Poblenou |  |
| 26 |  |  |  | Sant Martí de Provençals |  |
| 27 |  |  |  | la Verneda i la Pau |  |
| 28 |  |  |  | la Guineueta |  |
| 29 |  |  |  | la Prosperitat |  |
