# Supplementary figures and images for: Assessing the Distribution of Elderly Requiring Care: A Case Study on the Residents in Barcelona and the Impact of COVID-19
Source: Int J Environ Res Public Health. 2020 Oct 15;17(20):7486. doi: 10.3390/ijerph17207486 (PMC7602505; doi:10.3390/ijerph17207486)

**Figure S3. PCA of the neighborhoods**


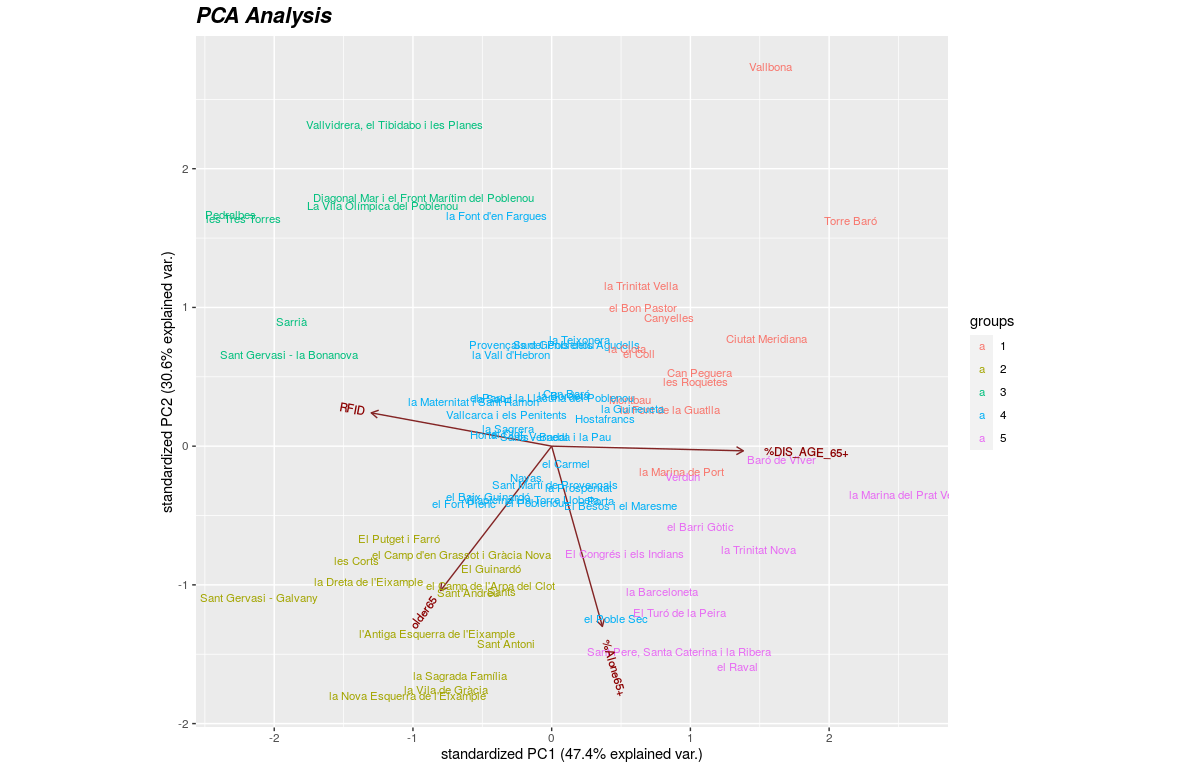

Supplement: Supplementary file 1 [file ijerph-17-07486-s001.zip › Figure S3.docx]

**Figure S4. Correlation matrix for data on 73 neighborhoods in Barcelona**


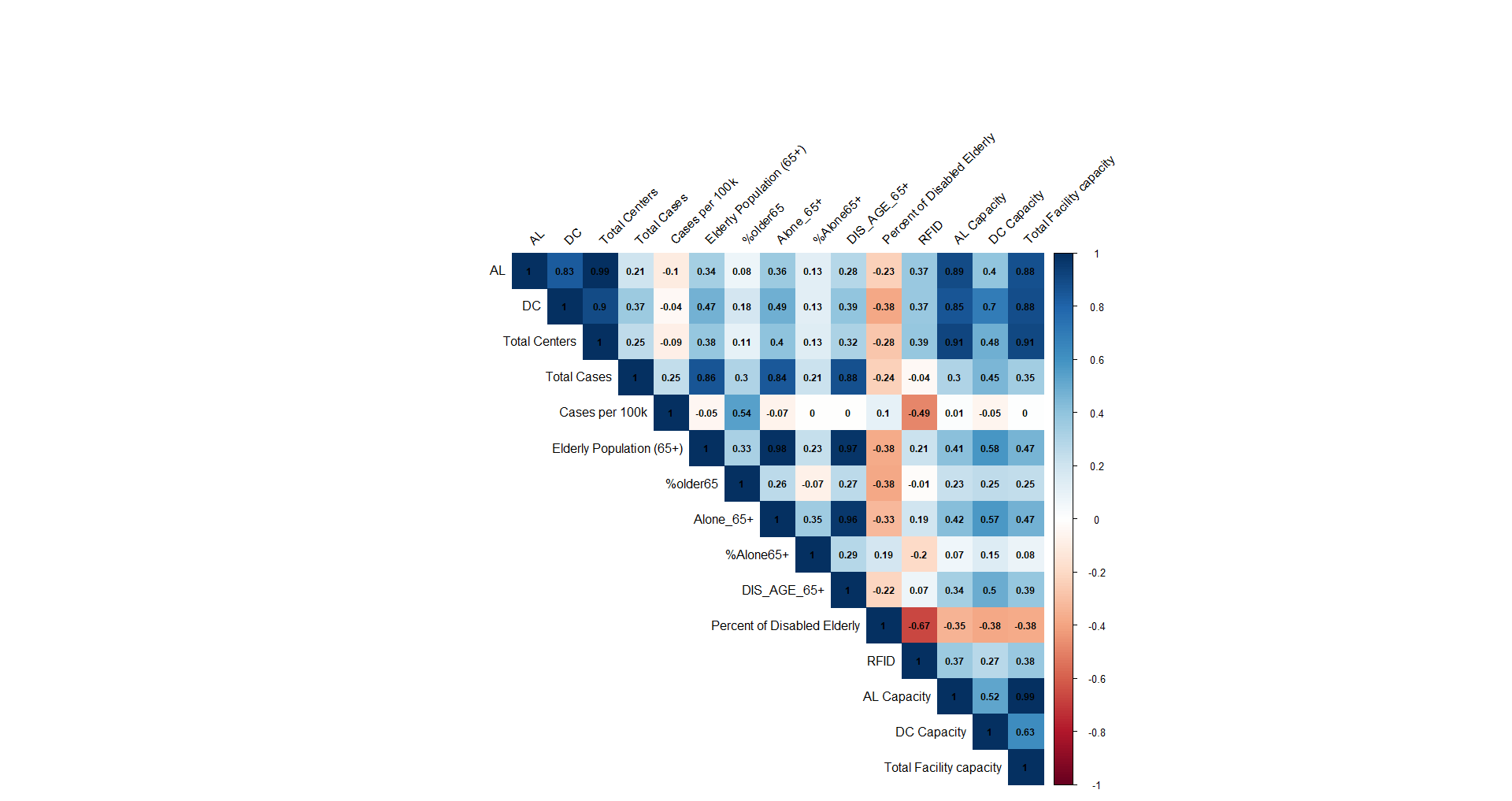

Supplement: Supplementary file 1 [file ijerph-17-07486-s001.zip › Figure S4.docx]
